# The Role of Satellite Cells in Skeletal Muscle Regeneration—The Effect of Exercise and Age

**DOI:** 10.3390/biology10101056

**Published:** 2021-10-18

**Authors:** Agnieszka Kaczmarek, Mateusz Kaczmarek, Maria Ciałowicz, Filipe Manuel Clemente, Paweł Wolański, Georgian Badicu, Eugenia Murawska-Ciałowicz

**Affiliations:** 1 Department of Physiology and Biochemistry, University School of Physical Education, 51-612 Wroclaw, Poland; pawel.wolanski@onet.pl; 2 Gynecology and Obstetrics Department, St. Hedwig’s of Silesia Hospital, 55-100 Trzebnica, Poland; mateusz.kaczmarek@yahoo.com; 3 Physiotherapy Faculty, University School of Physical Education, 51-612 Wroclaw, Poland; marysia.cialowicz@gmail.com; 4 Escola Superior Desporto e Lazer, Instituto Politécnico de Viana do Castelo, Rua Escola Industrial e Comercial de Nun’Álvares, 4900-347 Viana do Castelo, Portugal; Filipe.clemente5@gmail.com; 5 Department of Physiology, Gdansk University of Physical Education and Sport, 80-336 Gdansk, Poland; 6 Department of Physical Education and Special Motricity, Transilvania University of Brasov, 500086 Brasov, Romania; georgian.badicu@unitbv.ro

**Keywords:** satellite cells, muscle regeneration, myogenic regulatory factors, inflammation, exercise, age

## Abstract

**Simple Summary:**

Studies describing the effects of various forms of exercise and age on muscle regeneration were reviewed. Satellite cells are a heterogeneous group of cells that includes stem cells and skeletal muscle progenitor cells. Each skeletal muscle fiber has its own pool of satellite cells that remain inactive until the muscle is damaged. Minor damage within the cell membrane of muscle fibers is patched by fusing intracellular vesicles with the damaged sarcolemma. More severe muscle damage initiates a multistep regeneration process in which satellite cells play an essential role. The condition that initiates the cascade of reactions is the formation of inflammation at the structural discontinuity site, resulting in satellite cell activation. The multitude of reactions and pathways occurring during this process means that many different substances are involved in it and control it. Not all of them are well-understood yet. In parallel, the body’s own population of satellite cells is being rebuilt so that more fibers can be regenerated in the future. Athletes and the elderly are primarily at risk for muscle damage, and pathologies in muscle fiber regeneration cause serious diseases.

**Abstract:**

The population of satellite cells (mSCs) is highly diversified. The cells comprising it differ in their ability to regenerate their own population and differentiate, as well as in the properties they exhibit. The heterogeneity of this group of cells is evidenced by multiple differentiating markers that enable their recognition, classification, labeling, and characterization. One of the main tasks of satellite cells is skeletal muscle regeneration. Myofibers are often damaged during vigorous exercise in people who participate in sports activities. The number of satellite cells and the speed of the regeneration processes that depend on them affect the time structure of an athlete’s training. This process depends on inflammatory cells. The multitude of reactions and pathways that occur during the regeneration process results in the participation and control of many factors that are activated and secreted during muscle fiber damage and at different stages of its regeneration. However, not all of them are well understood yet. This paper presents the current state of knowledge on satellite cell-dependent skeletal muscle regeneration. Studies describing the effects of various forms of exercise and age on this process were reviewed.

## 1. History of Studies on Satellite Cells

The invention of the electron microscope in the 1930s sparked a revolutionary advance in research. The same was true for studies on mammalian muscle tissues. Specifically, satellite cells were first reported in the second half of the 20th century. In 1961, Alexander Mauro, a Rockefeller Institute scientist who studied frogs’ tibial muscles under an electron microscope, published a paper demonstrating the existence of mononuclear cells located peripherally between the sarcolemma and the basement membrane. He called them satellite cells [1]. That same year, in London, Bernard Katz also observed similar-looking cells during his studies on afferent nerve fiber endings in frog muscles [2]. Subsequent scientists tried to find out more about the structure, genesis, and role of these newly discovered cells. In 1966, Ishikawa made an unsuccessful attempt to describe the mSC structure [3]. Three years later, Kelly and Zacks showed that Ishikawa had confused satellite cells with connective tissue cells by calling them “fibroblast-like.” In the same paper, they described the histogenesis of the muscle tissues of rats’ intercostal muscles as an example [4].

In the following years, by labeling the tibialis anterior muscle cells of 14- to 17-day-old rats with a radioactive nucleoside (3H-thymidine), Moss and Leblond (1971) observed that the nuclei of multinucleated myofibers were derived from mSCs that were capable of mitotic divisions [5]. Observations of the mouse upper limb lumbrical cells made seven and 30 days after birth led to the conclusion that fewer and fewer satellite cells undergo fusion with muscle fibers as mice age. The rate of division of these cells did not decrease until the mice were three weeks old. In contrast, after the third week, mSC proliferation ceased, and they entered a quiescent state [6]. As early as during the discovery of satellite cells, Mauro postulated that they could participate in skeletal muscle regeneration [1]. This was eventually confirmed in 1975 by studies using phase-contrast microscopy [7].

In 1986, it was discovered that muscle injury causes satellite cells to exit the G0 phase and resume cell division [8]. Following these studies, researchers searched for factors that affect differentiation and the activation and deactivation of mitotic divisions of muscle satellite cells. One of the earliest agents studied was transforming growth factor-β (TGFβ). Studies have investigated its inhibitory effect on mSC differentiation [9]. The course of myogenesis in mammals has been established by observing mouse embryogenesis. During these observations, it was found that the first mSCs appeared under the basement membrane of the muscle on day 17 of fetal life [4].

Throughout the 1990s, many published studies described the role of various proteins in myogenesis, including myogenic regulatory factors (MRFs) and Pax7 in mSCs [10,11]. A 1990 paper by Bischoff addressed how the position of mSCs in the muscle affects their sensitivity to myogenic factors. It was shown that the niche in which satellite cells reside has an important influence on their performance [12].

In 2005, single myofibers with adherent mSCs were transplanted into irradiated mice that could not regenerate muscle. In this study, satellite cells were shown to have stem cell characteristics in that they were capable of regenerating their own population. Subsequent experiments helped understand the mechanism of mSC populations’ self-renewal process (asymmetric division) [13].

## 2. Pax7 Characterizes Inactive mSCs

Pax7 transcription factor belongs to a group of nine proteins encoded by *Pax* genes whose name comes from the DNA-binding domain called *“paired.”* These proteins have important functions during organogenesis, cell division, and differentiation. Over the centuries, the structures of these proteins have changed little, and their homologs are found in the proteomes of numerous animals, from simple organisms, such as nematodes, to insects, amphibians, fish, birds, or mammals [14]. The Pax7 protein is found in the cell nuclei of inactive satellite cells and in all human mSCs. This protein is responsible for regulating the division and differentiation of these cells [15]. Its effects include influencing myogenic determination protein 1 (MyoD1) and myogenic factor 5 (Myf5), which belong to MRFs [16]. The expression of the gene encoding this protein decreases in activated satellite cells [15].

In a study in mice that lacked the operational Pax7 gene (Pax7^−/−^), this factor was shown to maintain satellite cell populations after birth [16]. Although no significant differences in muscle thickness or appearance were observed in Pax7^−/−^ and wild-type mice, only a small percentage of the Pax7^−/−^-type mice reached adulthood. Survivors showed impaired growth and significant muscle tissue loss caused by a lack of functional mSCs [17].

## 3. Inflammatory Processes Involved in Muscle Regeneration

**Figure 1:**
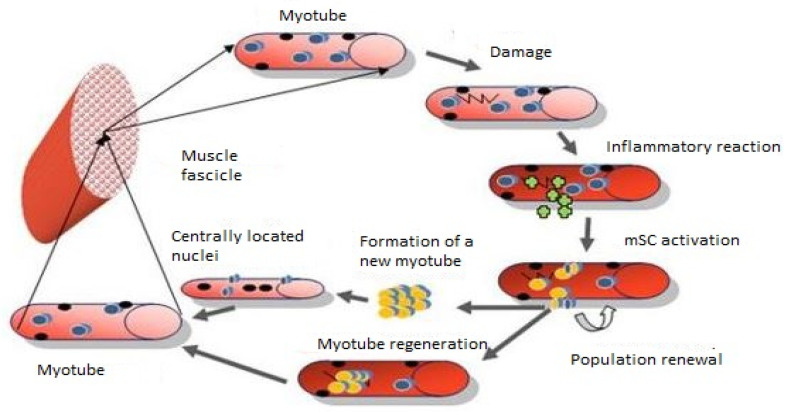
Schematic representation of the muscle fiber regeneration stages involving satellite cells. 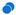
—quiescent mSC; 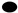
—nuclei; 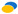
—active mSCs; 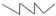
—myofiber damage; 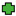
—inflammatory cells.

Mature skeletal muscle cells are characterized by high stability and are not subject to mitotic divisions [18]. Minor damage within the cell membranes of muscle fibers is patched by the fusion of intracellular vesicles with damaged sarcolemma. Caveolin 3, dysferlin, and one of two calpains (m or µ) are involved in this process. [19,20]. Severe muscle damage initiates a multistep regeneration process in which satellite cells play an essential role [21] (Figure 1).

Skeletal muscle fibers can be damaged by denervation, ischemia, or mechanical damage caused by (for example) the effect of high temperature. Intensive training, exposure to toxins, and genetic mutations that cause degenerative muscle diseases (including muscular dystrophies) may also cause this kind of damage [22]. Due to the disruption of the cell membrane of the muscle fiber, the concentration of Ca^2+^ ions increases rapidly inside the muscle fiber. This, in turn, activates proteolytic enzymes (calpain-calcium-dependent, non-lysosomal cysteine proteases) that digest the structural protein molecules of the damaged fibers. From damaged cells, the molecules of compounds normally present in the sarcoplasm enter the bloodstream. These compounds include creatine kinase, myosin heavy chain, lactate dehydrogenase, troponins, myoglobin, and beta-galactosidase [23].

Necrosis occurring in damaged muscle stimulates inflammatory processes—for example, the cascade activation of the complement system and an increase in the synthesis of proinflammatory cytokines [24]. Destroyed cells are phagocytosed by neutrophils and macrophages that migrate to the damage site [22,23]. The entire process begins with the stimulation of selectin family protein synthesis by tumor necrosis factor-α (TNF-α) and interleukin-1β (IL-1β). These substances promote the influx of neutrophils to the injury site, which takes 1–6 h [25]. The chemokines interleukin-6 (IL-6) and interleukin-8 (IL-8) also contribute.

Furthermore, neutrophil granulocytes secrete chemoattractants, resulting in the accumulation of two macrophage subpopulations at the injured muscle site [26]. The first of these is characterized by proinflammatory properties due to the secreted cytokines (TNF-α, IL-1β). The number of proinflammatory macrophages peaks approximately 24 h after fiber injury.

A defining feature of proinflammatory macrophages is the expression of the CD68 membrane protein, and their function is to remove the debris remaining after cell necrosis. The second subpopulation of macrophages is the anti-inflammatory group, which reaches its maximum concentration within two to four days after injury. Their properties are due to the secretion of cytokines that inhibit inflammatory processes—for example, interleukin-10 (IL-10). Unlike proinflammatory macrophages, they synthesize the CD163 protein (which is their marker) instead of the CD68 protein. The function of these macrophages is to protect newly created structures from proteolytic agents. In addition, macrophages stimulate satellite cell division [27]. Importantly, when the immune response is not triggered, satellite cells do not appear to be involved in regeneration [28].

## 4. Presence of MyoD1 and Myf5 Is Characteristic of Proliferating Satellite Cells

Satellite cells start dividing once anti-inflammatory scavenger cells appear in the muscle (i.e., on the second day after the injury occurs). Activation includes the satellite cells in the immediate vicinity of the resulting damage, as well as all cells adjacent to the damaged fiber. Activated mSCs migrate to the damage site, where the regeneration process continues [29]. The membranes of quiescent satellite cells contain the CD34 glycoprotein, which belongs to a family of adhesion proteins, the sialomucins. Immediately after satellite cell activation, there is a sharp decrease in CD34 production, allowing the molecules to adhere to each other less strongly and move more easily [30]. Researchers have observed that satellite cells can move not only within the same fiber but also between fibers and even between muscles. Such movement allows these cells to overcome basement membrane and connective tissue barriers [31].

Satellite cells undergoing division—called myogenic precursor cells (MPCs)—produce MRFs [21]. MRFs are characteristic of mSCs that have awakened from their quiescent state, and the expression of these factors occurs in an ordered sequence. In one study, MyoD1 and Myf5 were the first MRFs observed during the initial phase of mSC activation and division. In-depth analyses of single cells on the first day after injury show that some satellite cells begin to synthesize MyoD1 first, while others synthesize Myf5 first. On the other hand, the simultaneous expression of both factors occurs on the following day [10]. The appearance of both MRFs is necessary for satellite cells to exit the quiescent state. Cells in which no further MRFs are produced—and in which MyoD1 and Myf5 levels are decreased—revert to the pool of quiescent cells [32]. It is believed that MyoD1 expression determines MPC differentiation [33].

Megeney et al. [34] showed that cell division is not impaired in MyoD1^−/−^ mutant mice, though an accumulation of myoblasts was observed at the muscle injury site. However, these myoblasts do not differentiate into mature muscle cells. After examining them under an electron microscope, the authors did not report any abnormalities in their appearance [34]. However, Sabourin, et al. [35] demonstrated a different appearance of in vitro cultured mouse MyoD1^−/−^ cells. Compared to MyoD1^+/+^ cells, which were rounded, the mutant cells were flat and star-shaped [35]. In another study, another team of researchers showed that MyoD1^−/−^ muscles have branched muscle fibers, indicating an abnormal regeneration process within these myofibers [36]. These abnormalities may result from an imbalance between the satellite cells that stop proliferating, returning to the G0 phase of the cell cycle, and those that rapidly divide [33,34,35].

Asakura et al. [37] made an interesting discovery after injecting satellite cells into the tibialis anterior muscles of mice from the SCID/beige model that had been damaged by cardiotoxin (CTX). A significantly higher number of transplanted MyoD1^−/−^ cells survived in the regenerating muscle compared with the wild type. The difference was threefold after 24 h. Not only were there more of them, but they easily formed multinucleated myofibers and replenished the pool of quiescent satellite cells. The results of this experiment suggest that MyoD1 may regulate apoptosis, as a vast number of transplanted wild-type myofibers underwent programmed death during differentiation. The researchers tested this by subjecting the cells of both lines to UV radiation, which damages DNA. More apoptotic cells were shown in the wild-type MyoD1^+/+^ line than in fibers lacking this factor [37].

The characteristic phenotype of mice lacking Myf5 is muscle fiber hypertrophy, and proliferation is impaired in Myf5^−/−^ myoblasts. In contrast, Myf5 deletion does not affect the initial abundance of satellite cells. It is likely that the presence of Myf5 in the mSC determines which cell type it will differentiate into. In vivo studies have revealed that mSCs lacking Myf5 are more likely to differentiate into fibroblasts or adipocytes than into myofibers [38]. Additionally, it is postulated that Myf5 promotes the restoration of its own cell population by inhibiting the expression of MyoD1, thereby inhibiting further differentiation [31].

Previous studies suggest a distinct role of MyoD1 and Myf5 in muscle regeneration. MyoD1 has an overarching function in the initiation of cell differentiation, whereas Myf5 is involved in myoblast proliferation. The functions of these factors in adult myoblasts overlap with their roles during embryogenesis [39,40]. It can be speculated that the determination of muscle precursor cell development depends on whether MyoD1 or Myf5 expression is predominant. As an example of the dominance of MyoD1 factor expression, Myf5^−/−^ myoblasts showed early cell differentiation [21]. In contrast, the behavior of MyoD1^−/−^ myoblasts—in which increased proliferation and differentiation occurred with a significant delay—may serve as an example of a program path that is presumably followed by cells with Myf5 overexpression [32,33].

The expression of a protein that is crucial for cell differentiation—and, therefore, the regeneration of the MyoD1 proteins—is strictly controlled by the serum response factor (SRF). It binds to the DNA sequence recognized by SRF (SRE), which is located in the promoter of the myoD1 gene [41,42]. In proliferating myoblasts, low levels of MyoD1 are maintained by specific cyclin-induced reactions between cyclin-dependent kinase-4 and MyoD1 [43]. These compounds lead to the phosphorylation and subsequent degradation of the MyoD1 factor [44]. A decrease in the expression of genes, inducing cell division, results in the expression of myogenesis-enhancing factor-2 (MEF-2). As it competes with SRF for a binding site to the SRE sequence, it promotes MyoD1 expression and makes the cells enter the differentiation pathway [42]. By binding to gene promoters, MyoD1 facilitates the transcription of proteins that are characteristic of striated muscle tissue, such as M-cadherin. It also causes changes in the spatial arrangement of chromatin, thus allowing transcription proteins to subsequently bind to it [45].

## 5. Myogenin and Myf6 have Essential Roles in mSCs Differentiation

Early in cellular differentiation, MyoD1 initiates the formation of another MRF: myogenin. This occurs through the interaction of MyoD1 with enzymes that acetylate and deacetylate the gene promoter of this protein [46,47], and this process is controlled by Myf5 and MEF2 [31,47]. Myogenin expression is responsible for Pax7 inactivation in differentiating myoblasts [48]. This factor was also shown to enhance the formation of muscle cell-specific proteins whose expression was initiated by MyoD1 [49].

Myogenin is the only one among the four MRFs whose absence in fetal life leads to severe abnormalities in the development of muscle tissue, resulting in death soon after birth. The conclusion is that, just as the other factors compensate for each other’s effects, myogenin cannot be replaced by any of them. This situation changes immediately after birth. When gene encoding becomes inactive, this protein does not significantly affect the function of either myofibers or satellite cells. As a result, its role is taken over by other MRFs [50].

The last of the MRFs synthesized during differentiation is myogenic factor-6 (Myf6), the name of which is used interchangeably with muscle regulatory factor-4 (MRF4). Similar to myogenin, this MRF promotes cell division. Myf6 expression is regulated by MyoD1 and Myf5, and it is characteristic of terminal differentiation stages [36,46].

## 6. Fusion Is the Final Stage of Muscle Fiber Regeneration

MPC fusion is the final stage of muscle fiber regeneration following muscle damage. These cells either integrate with damaged fibers or fuse to form new syncytia (Figure 1). The latter process occurs in two stages. First, several myoblasts fuse to form a small *de novo* filament. Then, during maturation, more cells are recruited to the newly formed fiber, increasing the diameter of the spindle; this process is accompanied by increased contractile protein expression [21]. This process is complex and requires the participation of numerous molecules capable of reorganizing the structure of the intracellular cytoskeleton.

Over the years, it has been shown that the myoblast fusion process cannot take place without the involvement of transmembrane proteins responsible for cell-cadherin adhesion. Their action, in turn, is dependent on Ca^2+^ ions. Among the members of this family, M-cadherin plays the most important role in the formation of multinucleated myotubes during both embryonic myogenesis and regeneration [51,52]. This has been confirmed by numerous in vivo and in vitro studies.

This protein is found in mice and humans in most myofibers and satellite cells at different development stages. Muscle damage increases M-cadherin expression [52,53]. A significant reduction in M-cadherin expression was observed in injury-activated MyoD1^−/−^ satellite cells that could not integrate into fibers [33,34]. However, this protein is not irreplaceable, as the authors of the study in mice with disabled M-cadherin expression demonstrated. No abnormalities in muscle recovery after CTX injection were observed in these animals. The role of cadherin has been most likely compensated by other proteins from this family [54].

M-catherin is not the only adhesive protein responsible for the interaction of myoblasts. The most important adhesion proteins involved in the muscle fiber regeneration (without which the fusion of newly formed cells with existing fibers or with each other would not take place) are CD9, CD81, alpha-3, alpha-7, alpha-10, and beta-1 integrins, as well as vascular cell adhesion molekule-1 (VCAM-1) [55]. It is also likely that M-calpain, a Ca^2+^ ion-dependent proteinase, contributes to MPC fusion by reorganizing the cytoskeleton [56]. However, the mechanism of this process is still unknown.

One of the substrates for calpains is desmin, and mice with the desmin^−/−^ phenotype have been shown to have impaired regeneration and delayed cell fusion [57]. It has also been shown that satellite cells associated with the fiber differentiate into several myoblasts (equal to the number of nuclei of each satellite cell) within four to five days of fiber injury [58]. In mice, the first regenerated or newly formed muscle fibers are observed approximately five to seven days after injury [59]. These fibers are typically thinner than others, and their cell nuclei are centrally located. This changes during myofiber maturation as they move to the peripheral parts of the myotome [60].

In addition to muscle and satellite cells, connective tissue, an integral part of the muscle, is involved in mSC-dependent skeletal muscle regeneration. More specifically, these are mesenchymal stem cell-derived fibroblasts (collagen synthesis, organization of extracellular structures), adipocytes (which replace myofibers in some muscle diseases and during aging), and adipocyte and fibroblast progenitor cells [61].

## 7. Satellite Cell Self-Renewal

Satellite cells can divide in three different ways. First, symmetric division may result in two undifferentiated cells with stem cell characteristics. Second, two differentiating cells may be formed. Third, asymmetric division results in the formation of one undifferentiated and one differentiating cell [62]. The ability to reconstitute their own populations via undifferentiated progenitor cells is a distinctive feature of satellite cells. Presumably, maternal DNA strands are responsible for determining the fate of newly formed cells. One of them provides immortality [63].

To revise this hypothesis, Conboy et al. [64] damaged mouse muscles and injected them with a thymidine analog compound two days later, which the cells used during replication. Before the next cell division, they repeated this activity using a different thymidine analog. By analyzing mSCs extracted from muscle prepared with this method, the researchers found that selective inheritance of maternal DNA strands occurred in some of them. In addition, they observed that in most cases, the parental strands were found in cells that reproduced the mSC population [64].

**Figure 2:**
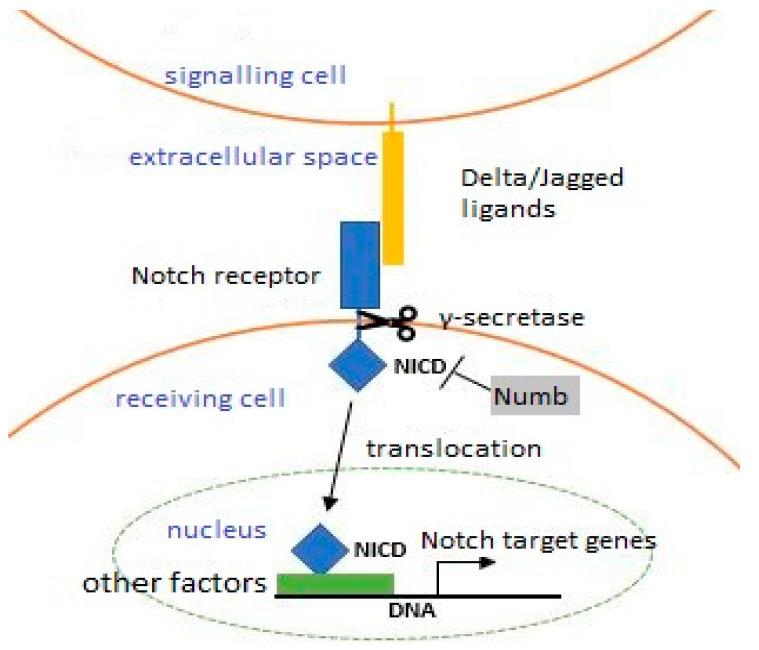
Notch signaling pathway (The description is in the text).

The mechanism that controls the proliferation of mSCs is the Notch protein signaling pathway (Figure 2). It is activated by Delta and Jagged ligands. Their interaction causes the Notch intracellular domain (NICD) to detach from the participation of γ-secretase. NICD enters the cell nucleus, where it induces the transcription of target genes with the participation of other factors. The Numb protein, a Notch receptor antagonist, is one of the compounds unevenly distributed between progenitor cells during asymmetric division, which may determine their fate. That cell exhibits a high concentration of Numb ligand and inhibits the Notch pathway, causing it to increase Myf5 factor and desmin levels and differentiate. The second Numb-depleted cell recreates a population of inactive satellite cells.

This was demonstrated in an ex vivo cell culture that contained myofibers and mSCs [65]. Studies on mice in which green fluorescence protein (GFP) synthesis depended on *Pax7* gene expression confirmed the effect of the Notch signaling pathway on its future. Notch pathway activity was high in cells that contained abundant GFP (Pax7+). GFP (Pax7-) detection decreased as Notch pathway activity decreased, whereas MyoD1 and myogenin expression increased as cells differentiated [66].

**Figure 3:**
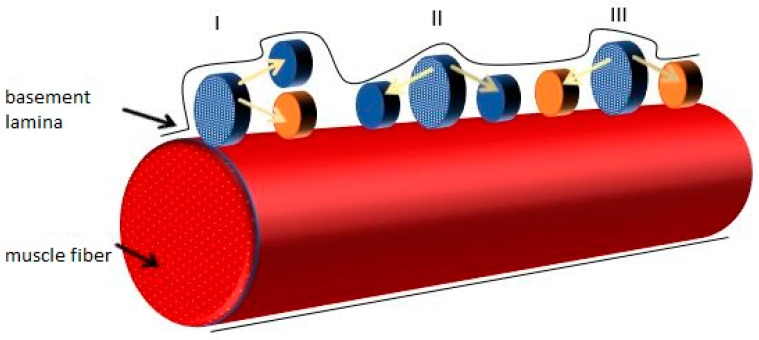
Renewing satellite cell populations. Blue cells—Pax7+/Myf5−; orange cells—Pax7^+^/Myf5^+^ cells; I—asymmetric division resulting in one differentiating cell (Pax7^+^/Myf5^+^) (orange cell), and another renewing a population of undifferentiated cells (Pax7^+^/Myf5^−^) (blue cell); II—symmetrical division resulting in two cells renewing the population of undifferentiated cells (2x Pax7^+^/Myf5^−^); III—symmetrical division resulting in two differentiating cells (2x Pax7^+^/Myf5^+^).

Whether a cell replenishes the mSC pool or undergoes differentiation is influenced by the division plane of the maternal cell. This has been observed in muscle cell cultures with mSCs attached to them. A progeny cell composition analysis for Myf5 factor revealed that one in ten Pax^+^ cells lacked Myf5 expression (Pax^+^/Myf5^−^). The majority of the remaining cells were Pax^+^/Myf5^+^ mSCs. Cells lacking Myf5 divide symmetrically and asymmetrically, but they do not differentiate and renew the mSC population. Furthermore, they do not lose contact with the basement membrane of the myofiber (Figure 3).

The researchers decided to use Pax7^+^/Myf5^+^ and Pax7^+^/Myf5^−^ cells in another experiment to test their biological properties. Each was separately transplanted into Pax7^−/−^ mice that were mSC−deficient due to impaired proliferation. Between 20 and 60 newly created fibers were formed when Myf5^+^ mSCs were administered, which is significantly more than in muscles into which Myf5^−^ mSCs were transplanted. In this case, the number of new fibers did not exceed 20. Administered Pax7^+^/Myf5^+^ were far more likely to differentiate than to participate in niche cell restoration. Additionally, when comparing Pax7^+^/Myf5^−^ cells with Pax7^+^/Myf5^+^, only the former could move in the muscle [13].

The Pax7 protein is also vital to the process of self-renewal; its high level is maintained both in inactive cells and proliferating ones, after which its expression decreases under the influence of myogenin, which is characteristic of differentiating cells [67]. Among the cells synthesizing the Pax7 protein and the MyoD1 factor, some did not reach the next stage (i.e., differentiation). A closer look using a thymidine analog revealed that the Pax7/MyoD1^−^ cells were the same cells that had normally expressed MyoD1 previously. MyoD1 activity decreased in these cells, and subsequent MRFs specific for cellular differentiation were not synthesized.

The researchers assumed that this cell lineage is responsible for self-renewal [67,68,69]. The same conclusions were reached by other authors, who additionally observed changes in the expression of nestin—a protein whose high level, as with Pax7, persists in inactive cells and those undergoing division, while decreasing during differentiation. Some researchers used transgenic mice in which GFP synthesis was coupled to nestin synthesis for this experiment. They found that nestin and Pax7 were present in population-renewing mSCs [40,68].

## 8. Satellite Cells and Physical Activity

After intense exercise—specifically, exercise that causes eccentric stretching of the muscle fibers—people may experience pain, increased tension, and limited mobility in the area of the exercised muscle. This phenomenon is referred to as delayed-onset muscle soreness syndrome (DOMS) [70]. Pain discomfort usually appears 12 to 48 h after exercise and reaches its maximum on the second or third day, after which it gradually subsides until the pain is completely gone. This lasts anywhere from a few days to a week [71]. Micro-damage occurs in the muscles due to intense exercise, constituting a signal to start the fiber regeneration process, which depends on satellite cells. The timing of pain coincides with inflammatory processes, which are the first stage of regeneration. The influx of neutrophils and (subsequently) macrophages to the injury site—along with the presence of inflammatory mediators—increases the sensitivity of neural tissue (specifically, nociceptors), which is thought to be the cause of pain [72].

The mitotic activity of mSCs can be stimulated by exercise in the form of strength (resistance) or endurance training (e.g., running). The changes in mSC activity and muscle appearance observed in subjects during a cycle of progressive running training indicate alternating fiber damage with regenerative processes [73]. This is reflected in the increased number of mSCs [74]. In an experiment in which subjects underwent regular exercise, an increase in mSCs was demonstrated as early as four days after the first exercise unit. Elevated levels of these mSCs were maintained in skeletal muscles throughout the entire training period. mSC levels started declining after completion of the exercises [75].

Human trials have demonstrated similarities and differences in the relationship between the type of exercise performed and the number of satellite cells. Therefore, clear conclusions cannot be drawn about these trials.

Resistance training is considered the primary method for increasing muscle mass. Nederveen et al. [76] analyzed the effects of a single bout of exercise and 16 weeks of resistance training on satellite cell activation. Satellite cell activity was detected when Pax7 and 4′, 6-diamidino-2-phenylindole (DAPI) (a marker used to stain cell nuclei) and/or Pax7, MyoD, and DAPI were observed together, as visualized by immunofluorescence.

An examination of the MyoD protein’s presence showed that, when tested before the 16-week training regimen, the number of cells with an activated MyoD gene (Pax7^+^/MyoD^+^) increased significantly 24 h after a single strength training session. In contrast, at the end of the 16-week experiment, significant differences were obtained after 24 and 72 h had passed. The increase was greater in the second case, suggesting that the organism had adapted to chronic exercise training, and indicating improved muscle regeneration processes due to a cycle of resistance training. The same study also found a positive effect from the training cycle on muscle capillarization. This improves the availability of ingredients necessary for muscle regeneration with the participation of mSC, thus improving the overall process [76].

The problems associated with satellite cell activity’s dependence on the type of exercise were also analyzed by Hyldahl et al. [77]. However, this study focused on comparing the effects of concentric and eccentric contraction on mSCs. The number of satellite cells increased in muscles subjected to an eccentric workout, but not in those subjected to a concentric workout.

A similar dependence on the type of contraction was observed in several parameters related to muscle damage, which were taken to be the expression of Xin (a skeletal muscle-specific protein whose concentration increases in proportion to the degree of muscle damage) and the disruption of extracellular matrix adhesion. The authors hypothesized that muscle injury-related changes in the extracellular matrix might regulate satellite cell activation and proliferation [77].

Differences in eccentric and concentric training were also studied by Farup et al. [78]. However, they broadened the issue to include protein supplementation. Twenty-two young men were divided into two groups of 11 participants each. Each followed the same resistance training program for 12 weeks, with each participant performing a concentric workout with one leg and an eccentric workout with the other (legs were chosen randomly to avoid the possible influence of single-leg dominance). The differences between the groups consisted of different dietary supplementation—the study group received 19.5 g of whey protein in combination with 19.5 g of carbohydrates, while the control group received a calorically equivalent placebo (39 g of carbohydrates).

Concentric training induced a significant increase in the number of satellite cells in the test and control groups and in both types of muscle fibers. Eccentric training induced an increase in mSCs in type I fibers in both groups; no significant change occurred in the number of mSCs in type II fibers, regardless of supplementation. There was also an increase in the ratio of mSCs to muscle cell nuclei in the group receiving protein supplementation, which was not the case in the control group.

The number of nuclei increased in type I fibers in all studied groups when the densities of cell nuclei were compared among different types of muscle fibers. Interestingly, concerning type II fibers, the group taking protein supplementation experienced an increase in the number of nuclei only for those who performed concentric training. Conversely, the increase occurred only in the group performing eccentric training in the placebo group.

These results indicate a clear dependence of mSC activity and cell nuclei accumulation on both types of resistance training and supplementation. This is especially true for type II fibers, as changes in type I fibers occurred regardless of the intervention type [78].

The reciprocal effect of different forms of exercise is also not insignificant. Babcock et al. [79] conducted a study in which a group of eight young men was subjected to two types of training: resistance training and mixed training (resistance training combined with aerobic exercise). Resistance training increased the number of satellite cells in the muscle fibers significantly more than mixed training. It was observed that aerobic exercise co-occurring in the training cycle with resistance exercises lessened the increase in the number of mSC in relation to the response after the resistance training itself. Therefore, the authors suggested that adding aerobic exercise to resistance training may reduce the muscle gains expected after resistance exercise [79].

Also, the ages of the people undergoing training can influence the results. Snijders et al. [80] studied the effects of a single resistance training session on satellite cell numbers and activation in relation to age. The number of satellite cells in type II fibers, relative to baseline values, in young and elderly subjects changed significantly 48 and 72 h after exercise, respectively. Myostatin content in both fiber types decreased 12, 24, and 48 h after exercise. However, at one and two days, the decrease was significantly higher among the younger subjects. The response of satellite cells to microdamage associated with completed muscle work was delayed with age. However, it was not impossible, confirming the presence of satellite cell activity in adult muscles and its potential involvement in muscle hypertrophy [80].

In an effort to compare the effects of resistance and endurance training on human skeletal muscles, Verney et al. [81] conducted a study involving elderly participants. Their upper bodies were subjected to resistance training, and their lower bodies were subjected to endurance training. The entire experiment lasted 14 weeks. Similar increases were observed in the number of mSCs in the deltoid muscle and the vastus lateralis muscle. However, as there was no repeatability in this experiment (because different muscles were tested), it cannot be assumed that the outcome was affected solely by the type of training.

A similar study in rodents also showed that changes in satellite cell numbers did not depend on the type of training, as both endurance and strength training increased the mSC pool [82]. The regenerative potential of the exercised muscle increased along with this pool. However, this does not always translate directly into muscle mass gains. Resistance training increases such gains [72,83], while endurance training can sometimes decrease them [84]. This correlates with a change in the number of muscle fiber nuclei [81,82], meaning that whether muscle mass increases or decreases does not depend entirely on the number of mSCs in the muscle.

Based on the effect of workout type on muscle mass gain and the problem of the occurrence of sarcopenia in the elderly, proper form and the reciprocal relationship between resistance and endurance training must be considered when selecting exercises for them. Inappropriately designed endurance exercise can have an effect opposite to the desired effect [85].

Shefer et al. [86] obtained different results when they studied the effects of endurance training on rats of different ages and both sexes. The number of satellite cells was higher in young animals of both sexes than in old animals (exercise and non-exercise groups, respectively). There was also an apparent sex difference, as young and elderly males had more satellite cells than the corresponding female age groups. More satellite cells were observed in animals subjected to exercise in each of the studied groups, with the increase being the greatest in young males [86]. It seems that a moderate intensity of endurance training is essential to increasing the number of mSCs.

The study by Murach et al. [87] showed that the impact of resistance training on the mSC population depended on the muscle fitness level. The number of satellite cells after resistance training in the untrained muscle increased at a much higher rate than after a cycle of endurance exercise. This demonstrates the evident influence of the cellular environment on the proliferative activity of satellite cells. It is also noteworthy that the reserve of inactive satellite cells was significantly higher after the 12-week endurance training cycle than before it. The authors hypothesized that resistance training programs presented a greater challenge for the untrained muscle at the cellular level. In a muscle accustomed to concentric endurance exercise, an increased reserve of satellite cells correlates with relatively little change in proliferative activity [87].

In addition to the type of exercise and age of the patients, the activity and number of stem cells depend on the broader environment. It appears that the type of muscle fiber may affect the number of activated mSCs that occur with it, although previous studies have not demonstrated conclusive results. Experiments in rodents comparing the satellite cell content of muscles predominantly composed of fast-contracting fibers with muscles composed mostly of slow-contracting fibers showed that type I fibers have more mSCs in untrained muscle than type II myofibers [82,86]. In contrast, no difference was found between type I and type II fibers when mSC content was tested in the vastus lateralis muscles of young, untrained humans [88,89,90]. However, after exercise, the number of mSCs increases significantly more in type II myofibers than in type I [82,91] owing to the observed hypertrophy of type II fibers after resistance training [85].

Cermak et al. [92] analyzed changes in the numbers of satellite cells before and after exercise in relation to individual types of muscle fibers in a group of young men practicing sports recreationally. There were no statistically significant differences in satellite cell content or activity in the type I and type II fibers in the biopsy taken before training. In the specimens obtained 24 h after training (when considering the mixed muscle type), there were no significant changes in the number of cell nuclei, the number of satellite cells, or their activity. However, when considering these values in the context of individual fiber types, the number and activity of satellite cells increased significantly in type II fibers (Delta like non-canonical Notch ligand 1 (DLK1) expression was taken as its determinant). Meanwhile, the values did not significantly change in type I fibers [92].

The number and function of mSCs also depend on other non-muscle cells. Mackey et al. [93] studied the interaction between satellite cells and fibroblasts in muscles damaged by electrostimulation-induced contractions. The numbers of fibroblasts and satellite cells increased at successive time intervals, and their mutual ratio changed. It was approximately 1.8 in favor of fibroblasts in the control group, increasing to 2.7 after 30 days. High levels of satellite cells after 30 days corresponded with a significant number of immature muscle fibers (labeled due to expression of neonatal or embryonic myosin).

The authors also cultured muscle cells in media containing different ratios of fibroblasts and satellite cells. The experiment showed that fibroblasts and mSCs interact not only on muscle fibers but also on each other. Specifically, satellite cells suppress the expression of certain collagen types, and fibroblasts regulate the maturation of satellite cells [93]. These findings are supported by a study in which the fibroblast pool was genetically reduced in mice, resulting in premature maturation of satellite cells [94]. These findings suggest the need to further investigate the interrelationship between satellite cells and fibroblasts, as well as the role they play in muscle recovery after exercise-induced damage.

Nielsen et al. [95] studied the effect of restricted blood flow in exercised muscles on mSC population. Twenty young, healthy men were divided into two groups: a study group that exercised with partial blood flow restriction (achieved by a compression cuff placed on the proximal aspect of the thigh) and a control group that exercised identically but without blood flow restriction. There was a three- to four-fold increase in Pax7^+^ expression in the study group when compared to their pre-workout values. The number of cell nuclei per muscle fiber also increased. Meanwhile, none of these values showed significant statistical changes in the control group.

Moreover, the muscle fiber cross-sectional area increased by 30–40% from baseline values. This increase was comparable in both groups in the biopsy taken on the eighth day of training, with the study group sustaining the increase on the third and tenth days after stopping training. Conversely, there was a marked decrease in the control group.

Having observed changes in the cross-sectional area of muscle fibers and in the number of cell nuclei, the authors hypothesized that the number of nuclei is the limiting factor for skeletal muscle hypertrophy, as it determines the volume of cytoplasm at which the cell will lose its ability to transcribe mRNA. However, the mechanism of such a significant effect of hypoxia on skeletal muscle hyperoxia itself remains unexplored [95].

The influence of physical activity on satellite cell population is multifactorial. It depends on type of activity, age of subject, protein supplementation, sex, and many other variables, some of them remaining unclear. Some recent papers oppose each other in terms of results, which suggests the need for further investigation.

## 9. Satellite Cells and Age

Aging processes occur in all living organism tissues. The changes that can be observed involve both morphology and function. Mass and contractile force decrease, and the muscle nerves in skeletal muscles degenerate. These processes start, and are reflected at, the level of reactions and transformations occurring in a single cell.

Rosenberg introduced the term “sarcopenia” in 1989, modifying it eight years later. Currently, sarcopenia is defined as a decrease in muscle mass and an accompanying decrease in muscle strength. Sarcopenia is a physiological sign of aging [96]. Studies show that it affects about 25% of people between the ages of 50 and 70 and 40% of people above the age of 80 [97]. The first symptoms are usually noticed after the age of 60. It is believed that the degeneration of α-motoneurons, resulting in the atrophy of entire motor units, is the cause of age-related reduction in muscle mass. These reductions are accompanied by demyelinating changes within axons. Furthermore, the numbers of nerve endings, vesicles, and receptors at synapses decrease.

Degenerative changes include motor end plates, which result in impaired and slowed nerve conduction, followed by denervation, which, combined with muscle cell atrophy, leads to permanent damage within the striated muscle tissue [98]. The denervated fibers are reinnervated by undamaged neighboring neurons. This process is called innervation, or reinnervation, and is often observed in older people. Unfortunately, it is not flawless. Occasionally, a small motoneuron starts innervating a fiber that is capable of rapid conduction, resulting in impaired muscle fiber recruitment during contraction and, consequently, impaired strength [99].

Experiments in rats have shown that, in many cases, the denervated fast-contracting fiber is reinnervated by motoneurons of slow-contracting units, causing their phenotype to change [100]. Histological observations have confirmed that large type II b fibers are the first to be damaged, and their mass is reduced significantly before the age of 80 years. Degradation of the slow-contracting units is also evident later. In cases where reinnervation cannot occur (or where denervation occurs too rapidly and the body cannot keep up with reinnervating the damaged areas), muscle cells are subject to atrophy and are replaced by fibrous cartilage tissue and adipose tissue [101]. These muscle changes, among other things, are responsible for the slower and less precise motions experienced by the elderly.

Testosterone also decreases with age, and such decreases are associated with muscle mass loss, reduced function, and decreased muscle strength. Testosterone hormone replacement therapy suppresses these phenomena, at least partially. The same is true for estrogen in women [102]. After the age of 30, the concentration of growth hormones in the human body begins to decline. In the elderly, low levels of these hormones increase myostatin expression, which inhibits mSC division. The regeneration of damaged muscle is also impaired, contributing to sarcopenia [103].

IGF-I levels in satellite cells also decrease in old age. Studies in which IGF-I was administered into atrophically changed aged rat muscles confirm that this factor increases the number of satellite cells and promotes muscle regeneration [104]. Observations are inconsistent regarding whether the number of satellite cells decreases with age.

Reductions in the number of mSCs have been observed in studies conducted in mice [105]. However, in another study, no significant differences were found in the number of mSCs when comparing the muscles of young and old rats [106]. The fact that different species of animals, different muscle types, and different age groups have been tested makes it difficult to draw conclusions from these experiments.

However, researchers agree that the proliferative potential of mSCs decreases with age [104]. This is related not so much to a decrease in their number as to changes in the microenvironment of aging muscles and, thus, in the niche specific for mSCs [107]. These changes are primarily associated with decreased concentrations of various factors that disrupt the regeneration process at various stages [104,105].

On the other hand, the sensitivity of mSCs to environmental signals also decreases. A group of scientists analyzed one of these signaling agents in 2003 and described the effect of age on Notch pathway activation. The muscles of young subjects, in which Numb protein levels were artificially increased, showed impaired muscle regeneration associated with early mSC differentiation.

The same regeneration pattern occurred in the muscles of elderly individuals. Based on this, the researchers ventured a step further: using an artificial ligand for Notch receptors in aged muscle, they obtained the same regeneration level as in young individuals. Per the above discussion, the number of Notch receptors does not change with age, and the impaired regeneration in aging muscles is associated with decreases in the number of Delta ligands [108].

Heterochronic parabiosis is a treatment that seems to support the hypothesis that the mSC environment, not the processes within it, has the greatest influence on skeletal muscle regeneration activity. This procedure involves fusing the circulatory systems of two organisms. When an old mouse was paired with a young individual, the former experienced a better regeneration of the previously damaged muscle than before the circulatory fusion. Conversely, the efficiency of the restoration process decreased in the young mouse. To ensure that regeneration processes in older mice were not related to whole cell transport, the researchers labeled young individuals ”GFP.” Satellite cells from old mice were labeled ”GFP^-^.” After the treatment, GFP detection did not demonstrate the presence of this protein in older mice. Hence, the environmental influences changed the mSCs’ fate. The same effects were obtained in the 1980s by researchers who transplanted part of the muscle of an older mouse into a young one and vice versa. However, they could not exclude the influence of intracellular processes in their experiments, which was finally achieved in 2005 [107].

Changes in the Wnt pathway—more specifically, the presence of its activators in the bloodstream—are observed in older subjects. Consequently, muscle progenitor cells lose their linear specificity with age. Therefore, instead of becoming myofibers, they increasingly start differentiating into fibroblasts or adipocytes, which contributes to the decreased functionality of the human muscular system [109].

Some researchers have traced the loss of function in aging mSCs to changes at the proteome level. The system that degrades damaged proteins includes lysosomes, autolysosomes, proteasomes, and chaperones. As people age, these structures no longer function as efficiently as they used to, causing residual degraded proteins to accumulate in cells, which often have toxic effects on those cells [110]. Cells that remain in a resting phase, including mSCs, are the most vulnerable to toxins.

The body’s aging process is often attributed to free radicals. Their destructive effects on mitochondrial membranes appear to increase with age as the efficiency of antioxidant mechanisms decreases. Studies have shown that type II fibers are more prone to free radical destruction than type I fibers, and damaged mitochondria are more frequently observed in them [111].

## Data Availability

Not applicable.
